# Progressive γδ T cell remodelling is associated with type-2 inflammation in eosinophilic chronic rhinosinusitis with nasal polyps

**DOI:** 10.3389/fimmu.2026.1857421

**Published:** 2026-07-15

**Authors:** Stjepan Grga Milanković, Mario Štefanić, Hrvoje Mihalj, Vjeran Bogović, Maja Jirouš Drulak, Željko Zubčić, Martina Mihalj, Stana Tokić

**Affiliations:** 1 Department of Otorhinolaryngology and Head and Neck Surgery, Clinical Hospital Centre Osijek, Osijek, Croatia; 2 Department of Otorhinolaryngology and Maxillofacial Surgery, Faculty of Medicine, Josip Juraj Strossmayer University of Osijek, Osijek, Croatia; 3 Department of Nuclear Medicine, Faculty of Medicine, Josip Juraj Strossmayer University of Osijek, Osijek, Croatia; 4 Department of Medical Chemistry, Biochemistry and Clinical Chemistry, Faculty of Medicine, University of Osijek, Osijek, Croatia; 5 Department of Dermatology and Venereology, University Hospital Osijek, Osijek, Croatia; 6 Department of Dermatovenerology, Faculty of Medicine, University of Osijek, Osijek, Croatia; 7 Department of Laboratory Medicine and Pharmacy, Faculty of Medicine, University of Osijek, Osijek, Croatia

**Keywords:** chronic rhinosinusitis, eosinophilia, inflammation, nasal polyps, γδT lymphocytes

## Abstract

**Introduction:**

Eosinophilic chronic rhinosinusitis with nasal polyps (eCRSwNP) is a type-2 inflammatory endotype characterised by tissue eosinophilia and mucosal remodelling. γδ T cells are tissue-resident lymphocytes involved in barrier immunity, yet their subset composition and relationship to inflammatory remodelling in eCRSwNP remain incompletely defined.

**Methods:**

γδ T cell subsets were analysed in nasal mucosa and polyp tissue from patients with eCRSwNP (n=21) and control subjects (n=10). Mononuclear cells isolated from nasal polyps and middle turbinate mucosa were examined by flow cytometry to quantify Vδ1^+^Vδ2^−^, Vδ1^−^Vδ2^+^ and Vδ1^−^Vδ2^−^ subsets. Targeted transcriptional analyses using RT-qPCR assessed expression of TRDV1, TRDV2 and type-2 inflammatory markers. Clinical indices included symptom scores, radiologic disease severity and histological eosinophilia.

**Results:**

eCRSwNP was associated with marked numerical and compositional remodelling of mucosal γδ T cells. Diseased tissue showed expansion of Vδ1^−^Vδ2^+^ and Vδ1^−^Vδ2^−^ subsets, whereas Vδ1^+^Vδ2^−^ cell numbers remained stable but declined proportionally within the expanding γδ compartment. These shifts followed a gradient from healthy mucosa to non-lesional tissue and nasal polyps and correlated with increasing disease severity, tissue eosinophilia and tissue-level type-2 inflammatory signatures.

**Discussion:**

Progressive γδ T cell remodeling across the sinonasal mucosa in eCRSwNP is associated with type-2 inflammatory activity, tissue eosinophilia and disease severity.

## 1 Introduction

Chronic rhinosinusitis (CRS) is a complex inflammatory disease of the nasal and paranasal sinuses, that manifests in two principal clinical forms: CRS with nasal polyposis (CRSwNP) and CRS without nasal polyposis (CRSsNP) (1). Although both entities share core symptoms, including nasal obstruction, congestion or nasal discharge, facial pressure, and olfactory dysfunction (2), they differ substantially in underlying immunopathology, tissue remodelling, disease course and response to therapy. Traditionally, CRS has been stratified into eosinophilic and non-eosinophilic endotypes, broadly corresponding to type 2 (T2) versus type 1/type 3 immune polarisation, with marked geographic and demographic variation in prevalence and clinical expression (3, 4). Within this framework, eosinophilic CRSwNP has long been recognized as a T2-driven disease, marked by dense tissue eosinophilia, elevated IgE levels, and high recurrence rates (5–7). Recent single-cell RNA sequencing (scRNA-seq) and spatial transcriptomic studies have substantially refined this view by revealing eosinophilic CRSwNP as a complex multicellular inflammatory ecosystem (8–12). High-resolution mapping revealed that altered fibroblasts (8), together with basal progenitor and tuft cell trajectories, contribute to extracellular matrix deposition and polyp formation (12), while macrophage-eosinophil recruitment circuits, dysregulated CD4^+^ and CD8^+^ T cell compartments, and transcriptionally diverse eosinophil (9) and myeloid (11) populations orchestrate and sustain type 2 inflammation (12). Comparative single-cell analyses further showed enrichment of Th2, M2 and cDC2 signatures in eosinophilic CRSwNP (10–13), alongside upregulation of canonical T2 mediators (*IL4, IL5, IL13, GATA3*), while non-eosinophilic CRSwNP and CRSsNP displayed neutrophil-skewed or Th1-biased immune architectures characterised by enhanced interferon (IFN) signalling and antigen presentation pathways. Within this highly interactive mucosal environment, unconventional lymphocyte populations, particularly γδ T cells, are strategically positioned at barrier sites, where they can mount rapid effector responses to epithelial stress signals, microbial products, and cytokines (14, 15). This localisation raises the possibility that γδ T cells participate in shaping or regulating the local inflammatory and tissue microenvironment in eosinophilic CRSwNP. However, due in part to their relative scarcity and transcriptional overlap with other lymphocyte populations, γδ T cells have been less consistently resolved in scRNA-seq datasets, leaving aspects of their subset composition and their potential links to eosinophilic inflammation and disease severity incompletely characterized. Human γδ T cells comprise heterogeneous, functionally distinct subsets defined by T cell receptor δ-chain usage, of which Vδ1 and Vδ2 are the most abundant (16). Vδ1^+^ γδ T cells preferentially localize to tissues and mucosal surfaces, where they have been implicated in epithelial surveillance, tissue adaptation, and cytokine production in chronic inflammatory settings (17). In contrast, Vδ2^+^ γδ T cells, most commonly paired with Vγ9, are enriched in peripheral blood and are classically associated with antimicrobial responses and type 1-biased immunity (18). Earlier immunohistochemical and flow-cytometric studies have reported increased γδ T cell infiltration in eosinophilic CRSwNP, with frequencies correlating with eosinophil burden, disease severity, and polyp recurrence (19–21). Functional observations on animal models and cell cultures further suggest that γδ T cells may contribute to T2 inflammation through cytokine secretion and cellular crosstalk with eosinophils (22) and B cells (23). Nevertheless, whether Vδ1 and Vδ2 subsets are differentially altered within eosinophilic CRSwNP tissue, and how this relates to nasal tissue remodelling, clinical outcomes and inflammatory disease indices, remains poorly understood. To address this gap, we integrated flow-cytometric profiling of nasal mucosa and nasal polyp derived γδ T cell subsets with targeted transcriptional analyses and clinical stratification of disease severity. Our findings reveal progressive, site-specific shifts in the Vδ1/Vδ2 landscape in eCRSwNP and link these changes to eosinophilia, inflammatory burden, and patient-reported disease severity.

## 2 Materials and methods

### 2.1 Study participants

A total of 31 patients were enrolled: 21 patients with eCRSwNP and 10 controls. All CRSwNP patients underwent functional endoscopic sinus surgery (FESS), whereas controls underwent other nasal procedures (septoplasty, rhinoplasty, inferior turbinate reduction, valvuloplasty). The diagnosis of CRS was based on clinical symptoms, endoscopic examination, and CT scans, in line with the European Position Paper on Rhinosinusitis and Nasal Polyps (EPOS) 2022 guidelines. All operations were performed by the same surgeon under general anaesthesia. Exclusion criteria included: age< 18 years, asthma, other respiratory or chronic diseases, prior sinus surgery, or corticosteroid/antibiotic use within one month before surgery. The study protocol was approved by the Ethics Committee of Osijek University Hospital (Ordered Number: R2-7990/2021) and the Faculty of Medicine in Osijek (Ordered Number: 2158-61-07-21-155). All participants provided written informed consent.

Disease-specific quality of life was evaluated using the SNOT-22 questionnaire, which includes 22 items scored from 0 to 5, giving a total score of 0–110, with higher scores indicating poorer outcomes. Endoscopic assessment of nasal polyposis was graded according to Malm (0–3), reflecting polyp extent from none (0) to complete obstruction (3). Radiologic staging was performed with the Lund–Mackay system, in which each sinus group (sphenoid, frontal, anterior and posterior ethmoid, maxillary, and ostiomeatal complex) was scored as clear (0), partly opaque (1), or completely opaque (2), and summed to provide a cumulative score.

### 2.2 Isolation of mononuclear cells from nasal polyps and middle turbinate tissue

Nasal polyps (NP) and middle turbinate (MT) mucosa were collected from eCRSwNP patients, and MT mucosa from control subjects. Tissues were placed in DMEM medium and transported to the DNA analysis laboratory within 1 hour for mononuclear cell isolation. Samples were minced into small fragments and incubated at 37 °C for 30 min on a LabRoller™ H5100 rotator in complete tissue culture medium consisting of DMEM supplemented with stable glutamine, 10% fetal bovine serum (FBS), 10 mM HEPES, 1% penicillin-streptomycin, 1mM sodium pyruvate, and 1 x non-essential amino acids. The resulting tissue suspension was further homogenized using a gentleMACS Dissociator (Miltenyi Biotec, Auburn, USA) and filtered through a 70-μm cell strainer to separate released cells from residual tissue fragments. Mononuclear cells were separated from erythrocytes and granulocytes by centrifugation (800 x g, 25 min, 25 °C, without brake) using Lymphoprep density gradient medium (Axis Shield, Oslo, Norway). The buffy coat was carefully collected, washed with phosphate-buffered saline (PBS) pH 7.5, pelleted (800 g, 10 min, 25 °C), and resuspended in 2 mL PBS buffer. Viable cell counts were obtained on a Luna automatic cell counter using trypan blue exclusion (cells were mixed 1:3 with trypan blue for counting purposes). On average, 5 × 10^5^ viable PBMCs were used per flow cytometry staining reaction in a final volume of 100 µL PBS.

### 2.3 Immunophenotyping of tissue γδ T cells

**Figure 1:**
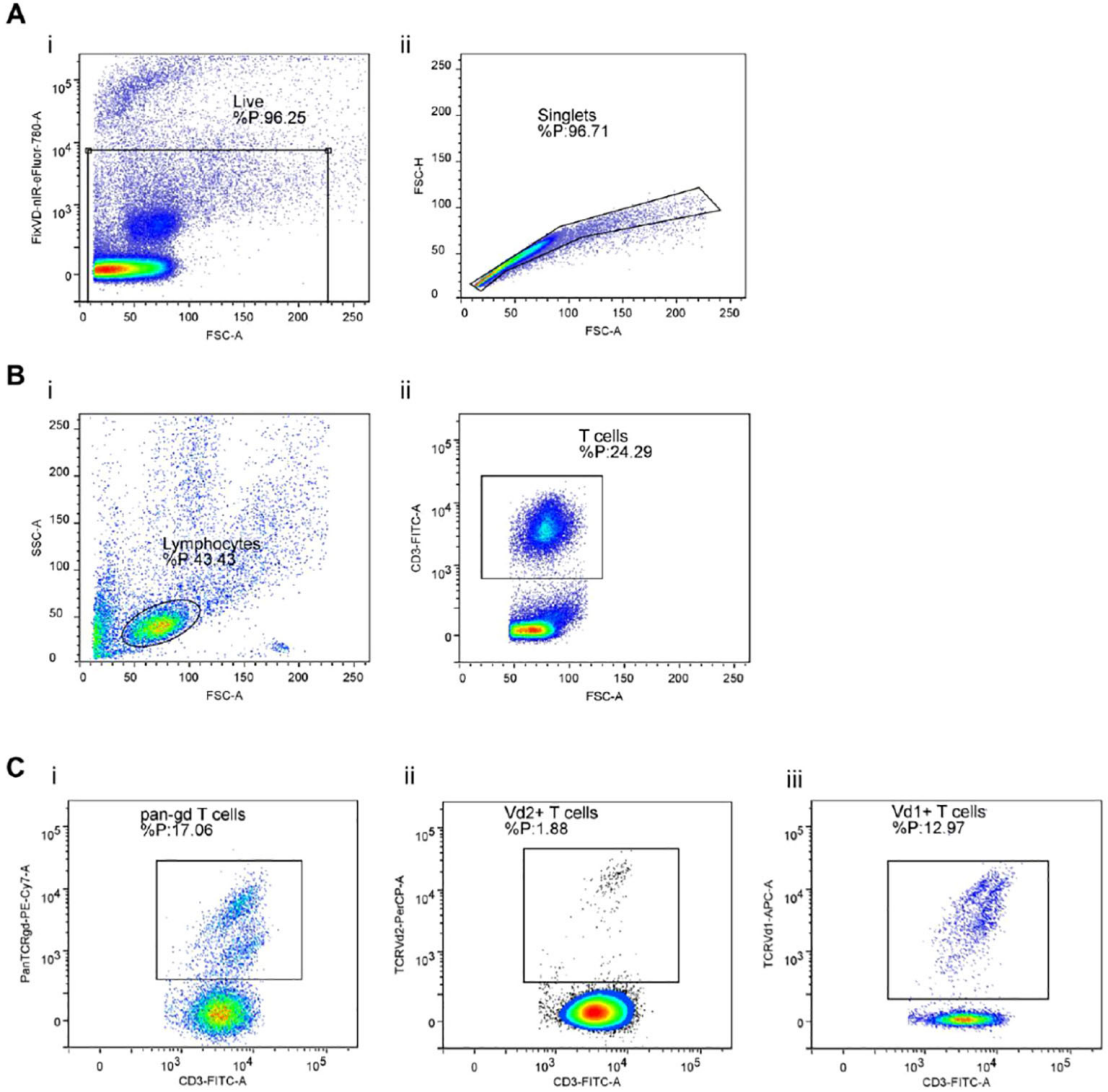
Gating strategy for identification and characterization of γδ T cell subsets by flow cytometry. Viable cells were first selected based on negativity for the fixable viability dye (FVD-negative population) **(Ai)**, followed by gating on single cells (singlets) using the forward scatter area (FSC-A) and forward scatter height (FSC-H) parameters to exclude doublets and aggregates **(Aii)**. In the subsequent step, the lymphocyte population was defined based on forward scatter area (FSC-A) and side scatter area (SSC-A), providing information on cell size and granularity, respectively **(Bi)**. The T-lymphocyte population was then identified based on the expression of the glycoprotein CD3 **(Bii)**. Further, the frequencies of total γδ T-lymphocytes **(Ci)** and the corresponding Vdelta 1 **(Cii)** and Vdelta 2 **(Ciii)** subpopulations were determined based on the expression of the pan-γδ TCR marker, the Vδ1 TCR chain, and the Vδ2 TCR chain, respectively. FVD, fixable viability dye; FITC, fluorescein isothiocyanate; PE-Cy7, phycoerythrin coupled with cyanine dye (Cy7); PerCP, peridinin chlorophyll protein.

The proportion of total γδ T lymphocytes and their subpopulations (Vδ1^+^Vδ2^-^, Vδ1^-^Vδ2^+^, Vδ1^-^Vδ2^-^) were determined in NP and MT mononuclear cells by flow cytometry (**Figure 1**). Dead cells were excluded using LIVE/DEAD™ Fixable Dead Cell Stain Kit (Thermo Fisher Scientific, Waltham, Massachusetts, USA). Nonspecific staining of Fc receptors (FcR) was performed with 5 μL of Human TruStain FcX™ Reagent (BioLegend, San Diego, California, USA). After 10 minute incubation at room temperature, cells were stained with the following antibody panel: CD3ϵFITC (clone UCHT1 gamma, donated by Prof. Peter Balogh, Faculty of Medicine, University of Pécs), TCRγδ PE-Cy7 (1:100, clone B1, BioLegend), TCRVδ1 APC (1:100, clone TS8.2, eBiosciences), and TCRVδ2 PerCP/CY5.5 (1:200, clone B6, BioLegend). PBS with 0.5-1% bovine serum albumin (BSA) and 0.1% sodium azide (NaN3) was used as a buffer. Compensation matrix was calculated with BD CompBeads Mouse Igκ/negative controls (BD Biosciences, San Jose, CA, USA) and single colour controls. Fluorescence-minus-one (FMO) controls were used to establish positivity thresholds and optimize gate placement for Vδ1 and Vδ2 populations. Data acquisition was performed on a BD FACSLyric™ flow cytometer (Becton Dickinson, San Jose, CA, USA) equipped with three lasers (blue Argon 488 nm, red HeNe 633 nm and violet 403 nm). Samples were anonymized and assigned sequential identification numbers prior to analysis, ensuring that flow cytometric acquisition and gating were performed blinded to clinical group allocation. Obtained data were analysed and visualised using FlowLogic v 11.0 (Inivai Technologies; Mentone; Australia).

### 2.4 Isolation and measurement of total RNA concentration

Total RNA was extracted from nasal mucosal mononuclear cells using Direct-zol™ RNA Microprep Kit (Zymo Research, Irvine, CA, USA), validated for RNA isolation from ≤ 10^5 cells. At least 5 x 10^4^ freshly isolated mononuclear cells were mixed with 300 μl TRIzol, combined with an equal volume of 100% ethanol and passed through filter columns by centrifugation (15,000 x g, 30 s). The filter-bound RNA material was purified by successive washes and eluted in 7 μl DNase/RNase-free water. RNA concentrations were measured with Qubit™ RNA HS Kit (Applied Biosystems) on a Qubit 3.0 instrument. All RNA samples were stored at -80 °C until further use.

### 2.5 cDNA synthesis and RT-qPCR experiments

cDNA was synthesised using PrimeScript™ RT Reagent Kit (Takara Bio, USA) in 20 μl reactions containing 4 μl PrimeScript™ buffer, 3 μl nuclease-free water, 1 μl oligo-dT primer, 1 μl random hexamer oligonucleotides (5 μM), 1 μl PrimeScript™ RT enzyme mix and 10 ng total RNA (c = 1 ng/μl). cDNA was diluted fivefold to 100 μl and used for RT-qPCR measurement of five target genes (*TRDV1, TRDV2, IL-4, IL-13, GATA3*) and 1 reference gene (*ACTB1*). The target transcripts were next amplified in a 10 μl volume reaction consisting of 0.5 μl of a custom or pre-made TaqMan gene expression assay (**Supplementary Table 1**), 5 μl TaqMan^®^ Universal PCR Master Mix (Applied Biosystems, Foster City, California, USA) and 4.5 μl cDNA. Amplification efficiency (81.9-113.3%) and linearity (R^2^ = 0.98-1.00) were confirmed using five fourfold serial dilutions of arbitrary cDNA standards. All RT-qPCR reactions were run in duplicate on a QuantStudio 5 real-time PCR instrument (Thermo Fisher Scientific, USA) and analysed with QuantStudio Design & Analysis Software v1.5.1.

### 2.6 Statistical data analysis

Categorical data are presented as ratios and absolute frequencies. Unless otherwise stated, observational data are summarized by the median and interquartile range. The Mann-Whitney test was used for two independent groups. For three and more independent groups, Van der Waerden test (24), and Van der Waerden’s many-to-one (control) *post-hoc* comparisons (single-step adjustment, PMCMRplus, v1.9.10) were used. Fisher’s exact test and the generalized Freeman-Halton version were applied to two-by-two and three-by-two contingency tables, respectively. Spearman’s rank test was used to assess univariate, pairwise correlations. Non-parametric partial correlations were tested using probability scale residuals (PResiduals, v1.0-1) (25). Cosine similarities were calculated using coop package v.0.6-3. A two-tailed P<0.05 was considered significant.

### 2.7 Data reduction

Patients were classified as having mild (<14, low, n=11) or extended (≥14, high, n=10) mucosal involvement based on the median Lund–Mackay CT score. Similarly, grades Malm 1 and Malm 2 were recoded as mild polyposis, as opposed to the more severe form, Malm 3. We also created multilevel predictors by combining categorical variables (sampling site × indexed LM score, sampling site × mucosal eosinophilia) into one parameter. This essentially stratifies the eCRSwNP samples according to disease severity and the extent of mucosal eosinophilia, allowing us to assess the homogeneity of the effects at different levels of the predictor variable. Log transformation (SNOT, IgE, and CRP levels) and winsorization (IgE levels) were used to stabilize the variance and re-normalize the distributions of the predictors when necessary. The presence of eosinophils in nasal tissue was binary coded (0/1, negative/positive). There was no missing data for any of the studied variables (flow cytometry arm). The figure and table legends contain all the relevant details.

### 2.8 Principal curve fitting

The one-dimensional representation of the compositional data (encompassing γδ subsets Vδ1^-^Vδ2^-^, Vδ1^+^Vδ2^-^ and Vδ1^-^Vδ2^+^) was generated using a concept of principal curve (26), a method of manifold learning based on nonparametric generalization of linear principal components (princurve v2.1.6 library, smooth spline). Essentially, principal curves are smooth one-dimensional curves that pass through the middle of an n-dimensional dataset, minimising the orthogonal distance from the points. For each sample, an ordering along the curve is provided by lambda parameter, representing the arc-length from the beginning of the curve. For this task, we adopted a recently proposed framework (27) starting with Euclidean distances and a principal coordinates analysis (https://zenodo.org/record/14967877). Ordination plots and PERMANOVA statistics (10,000 iterations) were generated using the vegan library (v2.6-4).

### 2.9 Regression analysis

Beta/binomial generalized linear mixed models were used to analyse compositional data or cell-type proportions (glmmTMB package v1.1.10 (28, 29)). Otherwise, a Gaussian response was modelled. Whenever feasible, we performed direct comparisons between polypoid and adjacent, non-polypoid eCRSwNP mucosa. As a result, most patients with eCRSwNP contributed more than one sample, leading to dependent (correlated) observations, which were modelled by fitting a random intercept (1|subject’s ID). Gene expression data were excluded from case-control comparisons due to limitations in cell numbers, and small number of observations.

As a rule, we fitted a full model with all the samples. If values of 0 and 1 were present in cell proportions, a *trafo* argument from the R package DirichletReg (v0.7-1) was used to transform the proportion data (30). Random-intercept models (starting from individual ID) were included where necessary (and possible) to account for correlated and hierarchical datasets. All presented models have successfully converged. The exact model specifications are available as Source Data. For the main result, we used second/order Akaike Information Criteria (AICs) to show that case-control status outweighs the contribution of demographic covariates (age and sex) to overall conclusions. For competing specifications, the best model (in terms of the trade-off between goodness-of-fit and model complexity) was selected by minimizing AICc (31–33). Near-equivalence of the competing alternatives was defined by ΔAICc< 2. The probability that a candidate model was the best in a set of competing specifications was determined using Akaike weights (AICcmodavg, v2.3–4 package). For description of each model, we refer the reader to the **Supplementary Table 2**. To investigate the effect of uncertainties on AICc rankings in sparse and unbalanced datasets, we also randomly resampled the data points (with replacement) from the given empirical distributions and refitted different explanatory models. We repeated this procedure until 10,000 successful iterations had been collected, resulting in 10,000 sets of best fits and their respective AICc scores. The confidence level was obtained by simply counting how many times the competing model was preferred with a smaller AICc score over the alternative one. Then, this number was divided by 10,000.

For linear mixed models the *ggeffects* (v2.3.1) package (34) was used to compute the marginal means and the adjusted predicted values of the response. Population-level predictions were corrected for Jensen’s inequality. This allowed us to visualize the effect of each predictor, which simplified the interpretation of the results. Pairwise contrasts were extracted using the *emmeans* package (v1.9.0) (35) and a Tukey’s adjustment. For a k-level factor, we retained only the comparisons which survived $\left(\begin{array}{c} k\\ 2 \end{array}\right)$ penalization. For the sake of completeness, we also provide the effect size on the logit scale (log-odds ratio with a 95% confidence interval, group-wise contrasts vs. controls) using a highly penalizing Tukey correction.

All data processing, analysis and visualization were performed using R4.3.1 (https://www.R-project.org). The complete list of all relevant R packages can be found in **Supplementary Table 3**. The codes supporting the results are available on reasonable request to the corresponding author.

## 3 Results

### 3.1 Subject characteristics

The study included tissue samples from 21 patients with eCRSwNP (aged 20–67 years, minimum–maximum) and 10 age-matched control subjects (20–51 years old) (**Table 1**). The control group comprised patients undergoing elective surgery for non-inflammatory structural nasal abnormalities, including nasal septal deviation, inferior turbinate hypertrophy, nasal valve insufficiency, and nasal pyramid deformities. Surgical procedures included septoplasty, rhinoplasty, nasal valve reconstruction (valvuloplasty), and inferior turbinate reduction. None of the control subjects had clinical, endoscopic, or radiological evidence of chronic rhinosinusitis with or without nasal polyps (CRSsNP or CRSwNP). Control middle turbinate (MNT) tissue samples and microbiological swabs were collected at the beginning of surgery, prior to any surgical manipulation, to ensure assessment of the native mucosal immune-cell composition. Eligibility criteria for controls included age ≥18 years, surgical indication for septal deviation, absence of nasal polyps, and provision of written informed consent. Individuals with chronic rhinosinusitis, previous sinonasal surgery, allergic rhinitis, physician-diagnosed asthma, active smoking or smoking cessation within the previous 6 months, recent upper respiratory tract infection, immunodeficiency, immunomodulatory treatment, systemic corticosteroid use within 8 weeks, or intranasal corticosteroid use within 4 weeks before surgery were excluded. Atopic disease was assessed by allergy history and skin prick testing or serum-specific IgE measurements to common aeroallergens, and only non-atopic individuals were included in the control group. Asthma was excluded based on clinical history and, where indicated, spirometry with bronchodilator reversibility testing. There was no significant difference in median age, SNOT-20 score, serum IgE, CRP levels (CRP: 0.5–3.3 mg/L, minimum–maximum), or the prevalence of allergic hypersensitivity (**Table 1**). The two groups, however, differed in gender distribution (**Table 2**). The control group was predominantly female (7 out of 10), while the affected group was mostly male (17/21). Altogether, 12 patients showed histologic evidence of eosinophilic infiltration at the time of the study. In particular, younger age and higher IgE levels were accompanied by higher eosinophil counts (Eo-positive/Eo-negative: 4/12 vs. 10/5, IgE<100 vs. IgE≥100 IU/mL, Fisher’s P = 0.031, **Table 2**). Women (0/4 vs. 11/6, low/high LM bin, F vs. M, Fisher’s P = 0.035) (27), younger individuals (Spearman’s R=–0.45, P = 0.048, n=21), and those sensitive to airborne allergens [13 (10–16) vs. 19 (15–21), neg. vs. pos. specific IgE test, n=15 vs. n=6, Mann-Whitney P = 0.032] had more extensive inflammation, as judged by Lund-Macay CT score. This probably indicates that severely affected individuals were more likely to seek tertiary help, and they did so much earlier. As a result, most patients were classified as having Malm grade 2 or 3 disease.

**Table 1:** Demographic characteristics of recruited subjects and flow cytometry data in collected tissue.

Attribute	Healthy controls	CRSwNP, MNT	CRSwNP, polyps
	(N = 10)	(N = 16)	(N = 19)
Age (yr)	42 [29, 47]	47 [38, 49]	47 [38, 53]
SNOT-20	29.00 [15, 40]	45 [28, 53]	45 [28, 50]
LM	NA	15 [10, 18]	14 [12, 18]
CRP (mg/dL)	1.3 [0.68, 1.83]	1.7 [1.18, 2.57]	1.5 [1.1, 2.4]
IgE	63 [26, 114]	105 [70, 283]	103 [55, 211]
Eosinophils (%)	0 [0, 0]	30 [0, 65]	20 [0, 65]
Vδ1^+^Vδ2^-^ (γδT)	0.72 [0.53, 0.75]	0.48 [0.25, 0.55]	0.36 [0.21, 0.62]
Vδ1^-^Vδ2^+^ (γδT)	0.09 [0.046, 0.133]	0.151 [0.074, 0.290]	0.114 [0.074, 0.203]
Vδ1^-^Vδ2^-^ (γδT)	0.18 [0.13, 0.29]	0.31 [0.22, 0.45]	0.42 [0.24, 0.57]
Vδ1^+^Vδ2^-^ (T)	0.023 [0.013, 0.057]	0.024 [0.017, 0.033]	0.023 [0.016, 0.031]
Vδ1^-^Vδ2^+^ (T)	0.003 [0.002, 0.005]	0.008 [0.003, 0.018]	0.009 [0.004, 0.012]
Vδ1^-^Vδ2^-^ (T)	0.009 [0.006, 0.017]	0.015 [0.012, 0.039]	0.023 [0.014, 0.045]
γδ (T)	0.037 [0.03, 0.08]	0.067 [0.046, 0.089]	0.065 [0.052, 0.084]
T (ly)	0.39 [0.36, 0.46]	0.427 [0.325, 0.657]	0.554 [0.333, 0.649]

SNOT-20, sinonasal outcome test; LM, Lund Mackay; CRP, C reactive protein; NA, not available; MNT, middle nasal turbinate.

**Table 2:** Clinical attributes of recruited subjects.

Attribute	Healthy controls	CRSwNP, MNT	CRSwNP, polyps
	(N = 10)	(N = 16)	(N = 19)
Sex			
F	7 (70.0%)	4 (25.0%)	3 (15.8%)
M	3 (30.0%)	12 (75.0%)	16 (84.2%)
LMIndexed			
c	10 (100%)	0 (0%)	0 (0%)
high	0 (0%)	8 (50.0%)	9 (47.4%)
low	0 (0%)	8 (50.0%)	10 (52.6%)
MalmIndexed			
c	10 (100%)	0 (0%)	0 (0%)
High (Malm 3)	0 (0%)	5 (31.3%)	9 (47.4%)
Low (Malm 1 + 2)	0 (0%)	11 (68.8%)	10 (52.6%)
InhAllerg			
neg	6 (60.0%)	11 (68.8%)	13 (68.4%)
pos	4 (40.0%)	5 (31.3%)	6 (31.6%)
NutrAllerg			
neg	10 (100%)	14 (87.5%)	16 (84.2%)
pos	0 (0%)	2 (12.5%)	3 (15.8%)
Eosinophils			
neg	8 (80.0%)	6 (37.5%)	8 (42.1%)
pos	2 (20.0%)	10 (62.5%)	11 (57.9%)

SNOT-20, sinonasal outcome test; LM, Lund Mackay; CRP, C reactive protein; Sex (F- female, M-male), c – healthy control; LMIndexed, Lund Mackay index; InhAllerg, inhalant allergene test; NutrAllerg, nutritive allergene test; MNT, middle nasal turbinate.

A subset of donors who provided sufficient tissue material for RT-qPCR analysis is described in **Table 3**. Overall, the polyps (n = 15) provided more data points than the controls (n = 4) and the MNT samples (n = 6) combined, corresponding to significantly thinner lining of the MNT mucosa compared to the polypoid tissue.

**Table 3:** qPCR subset, clinical features.

Attribute	CRSwNP, MNT	CRSwNP, polyps
	(N = 6)	(N = 15)
Sex		
F	2 (33.3%)	2 (13.3%)
M	4 (66.7%)	13 (86.7%)
LMI		
high	3 (50.0%)	7 (46.7%)
low	3 (50.0%)	8 (53.3%)
MalmIndexed		
High (Malm 3)	3 (50.0%)	6 (40.0%)
Low (Malm 1 + 2)	3 (50.0%)	9 (60.0%)
InhAllerg		
neg	4 (66.7%)	11 (73.3%)
pos	2 (33.3%)	4 (26.7%)
NutrAllerg		
neg	6 (100%)	12 (80.0%)
pos	0 (0%)	3 (20.0%)
Eosinophils		
neg	3 (50.0%)	6 (40.0%)
pos	3 (50.0%)	9 (60.0%)

F, female; M, male; LMI, Lund Mackay index; InhAllerg, inhalant allergen test; NutrAllerg, nutritive allergen test; MNT, middle nasal turbinate.

### 3.2 eCRSwNP is associated with numeric and compositional remodelling across the local γδ subpopulations

**Figure 2:**
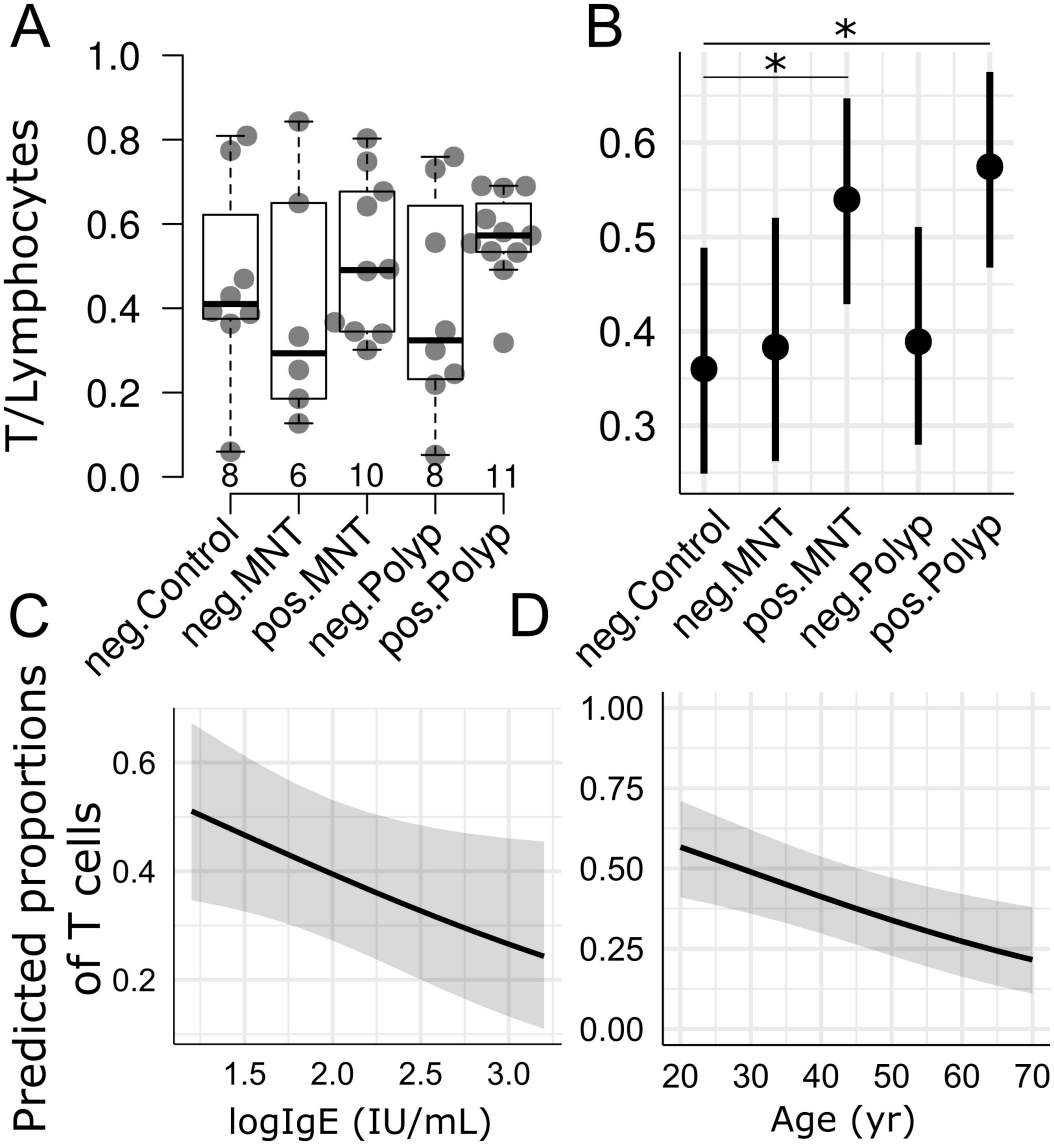
T cell frequencies, stratified by the presence (prefix: pos) or absence (neg) of eosinophil infiltration in different mucosal locations. MNT and Polyp designate individuals with eCRSwNP. Two eosinophil-positive control samples were excluded from the subgroup analysis. **(A)** Raw cytometry data; boxplots are defined by medians and their respective interquartile ranges (IQRs). Vertical lines extend to ±1.5 IQR. **(B–D)** Beta-regression, linear mixed model. The predicted proportions of T cells are shown after accounting for sample-relatedness (modelled as random intercept, 1|subject’s ID), age **(C)**, and log-transformed serum immunoglobulin E (IgE), and log-transformed serum immunoglobulin E (IgE) levels **(D)**. Shaded ribbons denote the uncertainty of the marginal effect rather than the width of true distribution. The group-wise sample size is available in Panel A. An asterisk (*) denotes nominal P< 0.05 vs. eosinophil-negative controls. No tissue was significantly different after global correction (5-level Tukey test, 10 pairwise comparisons). Source data are provided in the Source Data file.

Flow cytometric analysis was performed on 35 tissue samples from affected individuals (19 nasal polyps and 16 MNT specimens), yielding 14 paired samples (**Table 1**). In healthy controls, only MNT mucosa was analysed (n=10). In comparative quantifications, the percentage of mucosal T cells among lymphocytes did not differ strongly from healthy MNT samples (**Figure 2**). Apparently, younger donors had a higher frequency of mucosal T cells than older ones; in addition, higher serum IgE levels were associated with lower T cell numbers (**Figure 2**; **Supplementary Table 4**).

**Figure 3:**
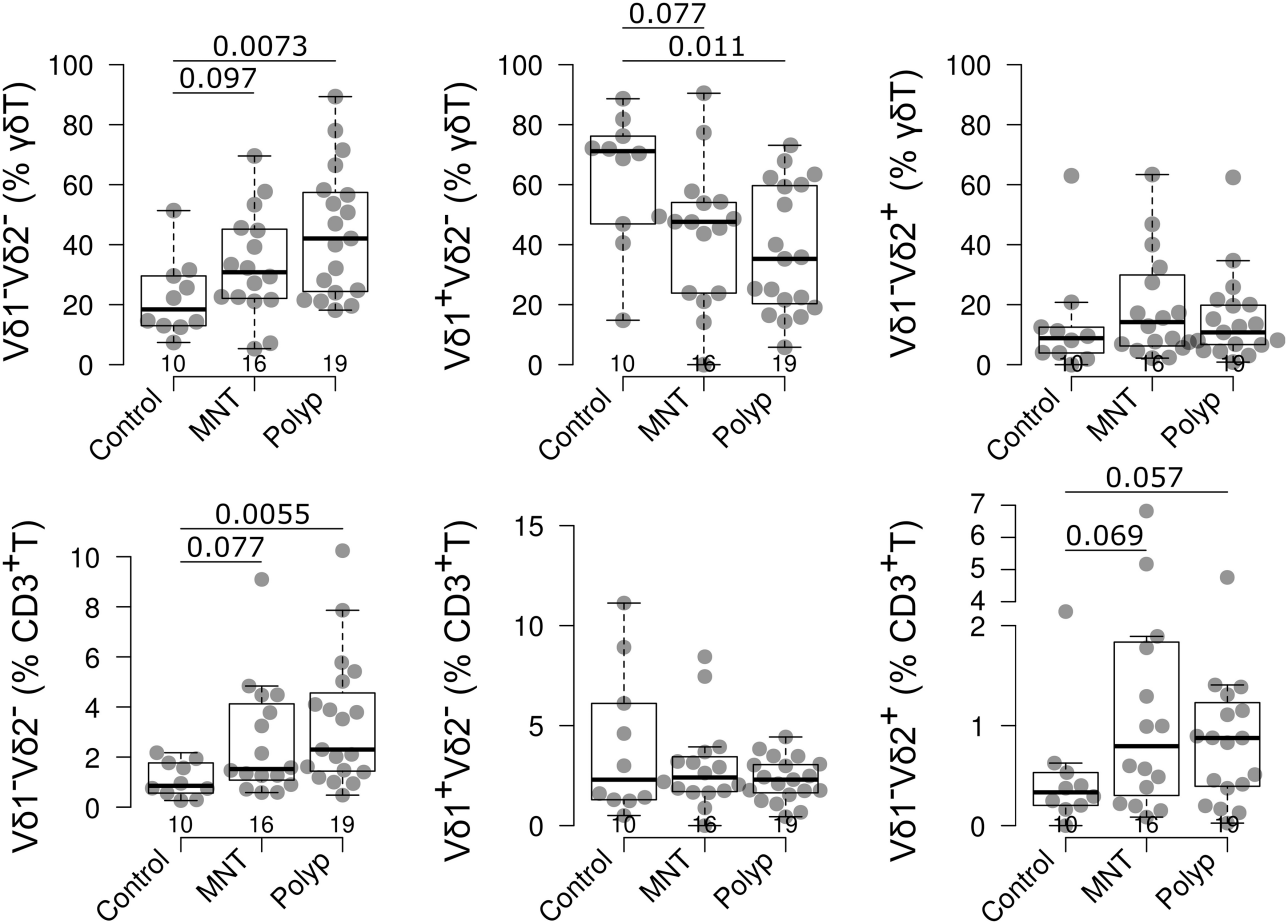
Raw cytometric data, stratified by sampling site and case-control status. The percentage occupied by each cell fraction among the γδ (upper row) and CD3+ T cells (bottom row) is shown. Mann-Whitney test (P-value), pairwise comparisons *vs.* controls. MNT medium turbinate mucosa, affected individuals. Note that the MNT and Polyp groups are not independent; hence, the nonparametric tests only provide an exploratory study of qualitative differences in observable characteristics. Box plots represent the median (center line), interquartile range (box), and whiskers extend to the minimum and maximum values within 1.5 × IQR. For simplicity, all comparisons with two-tailed P≥0.1 are omitted from presentation.

**Figure 4:**
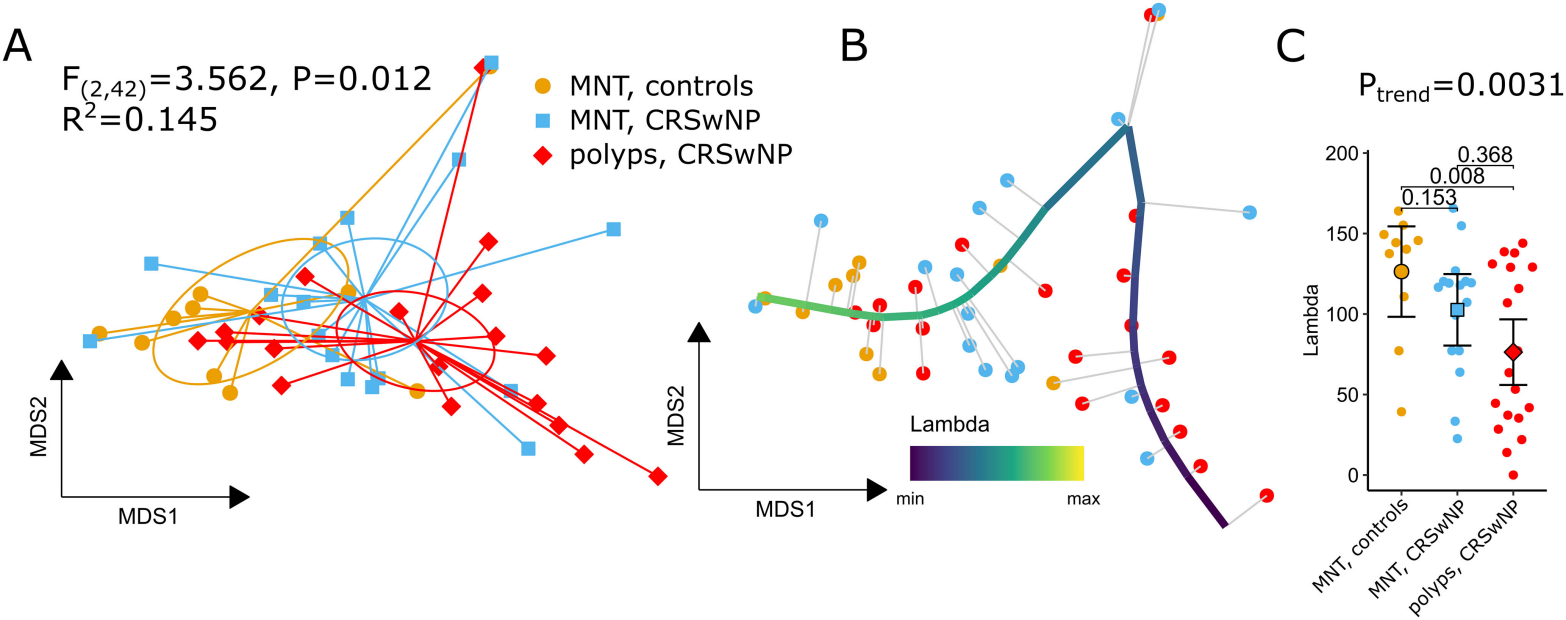
Multidimensional scaling (MDS), the first two components (MDS1 and MDS2) were used for visualization. The analysis was performed using percentage contributions of Vδ1+Vδ2-, Vδ1-Vδ2+, and Vδ1-Vδ2- subsets to total γδ T cell number. **(A)** Ordination plot, showing significant separation of the groups. Each element, colored by its group membership, corresponds to one sample. P-value indicate group centroid comparisons using a permutational analysis of variance (n=10,000). 95% confidence level was used to construct the ellipses. F-statistics, R² represents the proportion of variance explained. **(B)** The nonlinear principal curve show individual sample projections along the compositional gradient. The parameter lambda (colored ribbon) quantifies a sample’s position along the principal curve by measuring the corresponding distance from a defined starting point. **(C)** The lambda parameter distribution across the groups. Error bars representing the adjusted marginal means and their 95% confidence intervals. The P-values correspond to the linear trend test (orthogonal polynomial contrasts) and the Conover’s *post-hoc* test for pairwise comparisons. Note the visibly bimodal grouping of polyps. Here, the smaller lambda corresponds to a greater distance from the control samples.

The total proportion of γδ T cells and their Vδ1|Vδ2 chain usage were consistent with a nasal (12) or mucosal origin (15). Overall, the nasal mucosa preferentially hosted Vδ1^+^Vδ2^-^ and Vδ1^-^Vδ2^-^ cells (**Figure 3**; **Table 1**). The number of Vδ1^-^Vδ2^+^ cells was often dwarfed by Vδ1^+^Vδ2^-^ and Vδ1^-^Vδ2^-^ numbers, but this varied by case-control status and disease severity. Broadly, an ordered gradient of compositional changes progressing from normal mucosa through non-lesional MNT tissue to nasal polyps (**Figure 4A**) was revealed by summarizing the data into a principal curve (**Figures 4A, B**). Specifically, γδ T cells from non-lesional MNT mucosa tended to project closer to control MNT tissue along the principal curve, showing much tighter distributions of lambda scores (λ) compared to cells from polyps (**Figures 4A–C**). Polyps showed greater heterogeneity in bulk Vδ chain composition (**Figure 4C**).

By contrast, age and sex alone had little explanatory power for γδ T cell data, as indicated by strong preference for the null (constant) model over the linear combination of the two in a bootstrap analysis (**Supplementary Table 5**). Similarly, the case-control status and topography confidently outperformed the age+sex model, ruling out a major impact of demographic differences on overall conclusions (**Supplementary Table 5**). A detailed overview is provided in a companion paper that quantifies the effects of covariates in non-eoCRSwNP (36); here, we simply focus on the case-control comparisons by integrating out the effects of nuisance variables.

### 3.3 Vδ1^-^Vδ2^-^ and Vδ1^-^Vδ2^+^ subsets are differentially altered in polypoid and non-polypoid eCRSwNP mucosa

**Figure 5:**
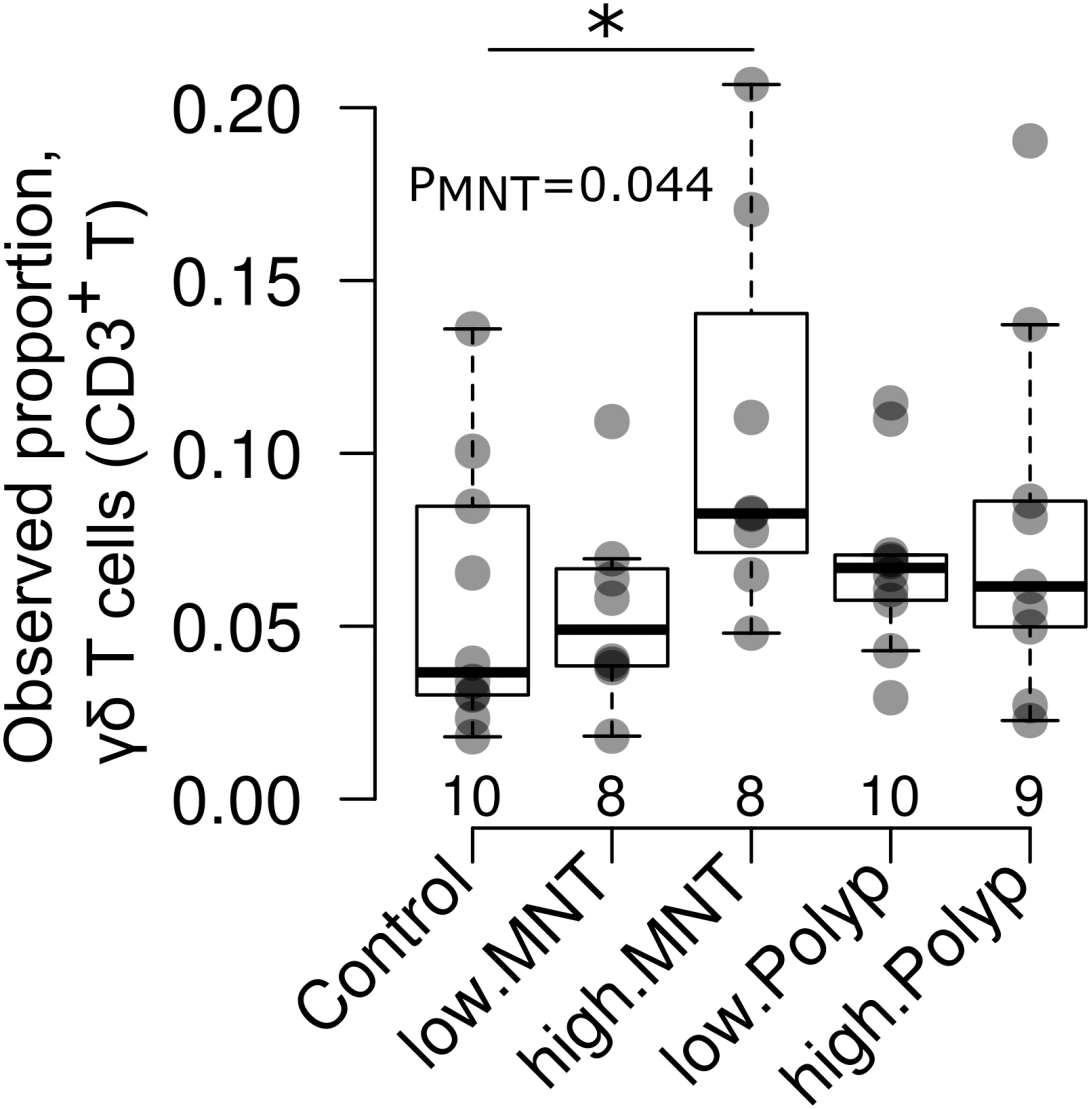
The frequencies of total γδ T cells (raw data, relative to T cells), split by case-control status, mucosal location, and extent of inflammation (binary Lund-Mackay score, prefix: low<14, high≥14). MNT and Polyp designate individuals with eCRSwNP. Van der Waerden test (global P-value) for independent groups (MNT *vs.* controls, Polyp *vs.* controls), followed by Van der Waerden’s many *vs.* one (control) *post-hoc* comparisons (single-step adjustment). An asterisk denotes a significant difference compared to controls (*P< 0.05, **P< 0.01, ***P< 0.005). For simplicity, all non-significant comparisons are omitted from display.

**Figure 6:**
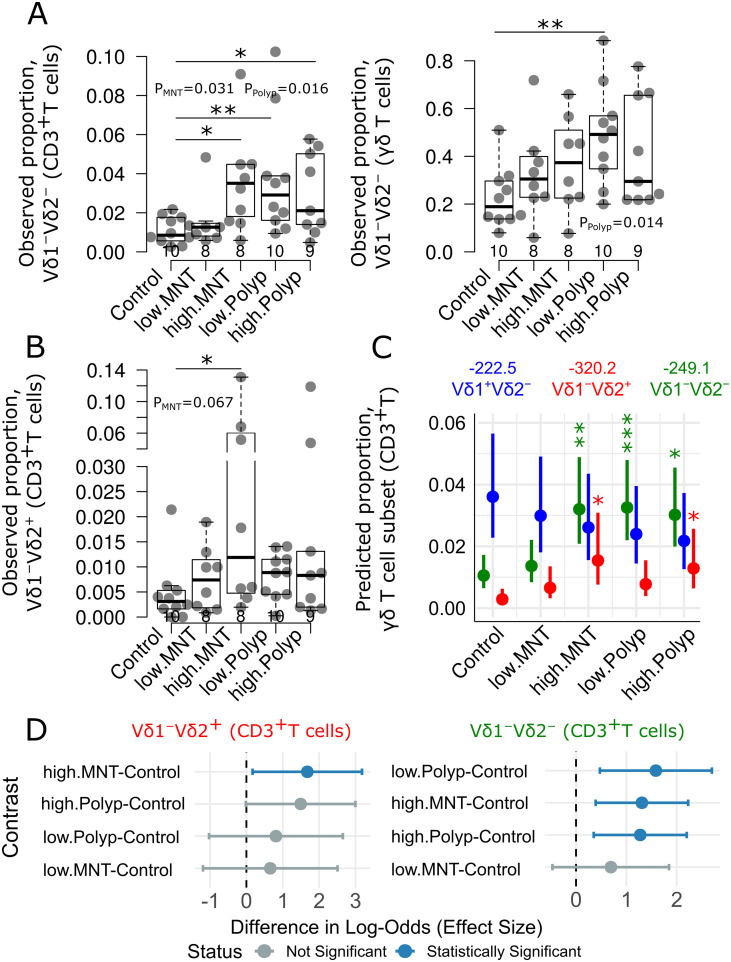
The frequencies of γδ T cells subsets [raw data, **(A, B)**], split by case-control status, mucosal location, and extent of inflammation (binary Lund-Mackay score, prefix: low<14, high≥14). MNT and Polyp designate individuals with eCRSwNP. **(A)** Vδ1-Vδ2- cells, **(B)** Vδ1-Vδ2+ cells. Van der Waerden test (global P-value) for independent groups (MNT *vs.* controls, Polyp *vs.* controls), followed by Van der Waerden’s many *vs.* one (control) *post-hoc* comparisons (single-step adjustment). **(C)** Beta-regression, linear mixed model. The predicted proportions of cells are shown after accounting for sample-relatedness, age, and sex (5-level Tukey’s *post-hoc* comparisons). An asterisk denotes a significant difference compared to controls (*P< 0.05, **P< 0.01, ***P< 0.005). For simplicity, all non-significant *post-hoc* comparisons are omitted from display. For visual clarity, the data points corresponding to different color-coded cell subsets (horizontal label) are slightly offset horizontally. The colored numbers correspond to the second-order corrected Akaike information criteria for each model. The group-wise sample size is available in Panel **(A, D)** The forest plot depicting the effect size (log-odds ratio and 95% confidence interval with Tukey’s correction) for the selected comparisons from Panel **(C)** Statistically significant differences are indicated by a blue color (95% confidence intervals excluding zero).

**Figure 7:**
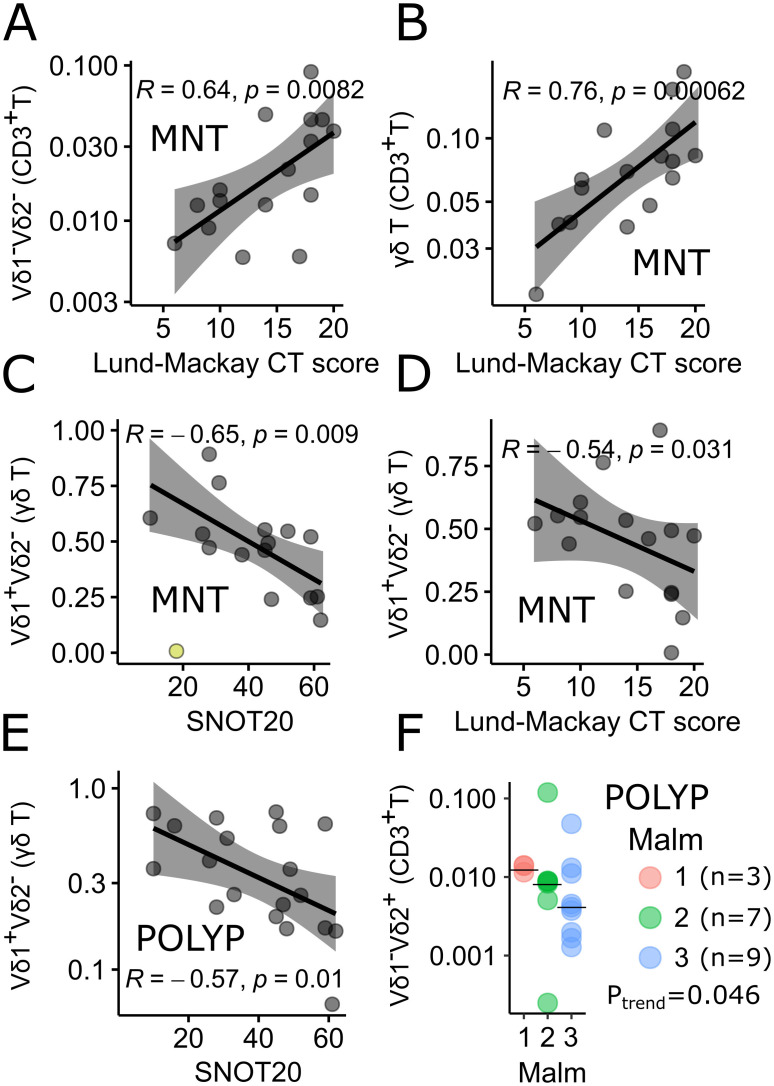
The scatterplots show the relationship between different cell type proportions **(A–E)** and measures of disease severity (Lund-Mackay CT score and Sino-Nasal Outcome Test-20 [SNOT-20]). The relations are plotted separately for each sampling site (polypoid and medium nasal turbinate (MNT) mucosa, eCRSwNP). One dot corresponds to one donor. The solid black line represents the least squares fit, the shaded ribbon corresponds to 95% confidence interval. R and P-values belong to Spearman’s rank correlation test. **(C)** One possible outlier was excluded from the fitting (the yellow dot). **(F)** The dot plot shows the distribution of cell type proportions in polypoid mucosa according to the Malm endoscopic score. The horizontal line represents the median value for each group. The P-value corresponds to the linear trend test (orthogonal polynomial contrasts). The Y-axes **(A, B, E, F)** are log-scaled.

**Figure 8:**
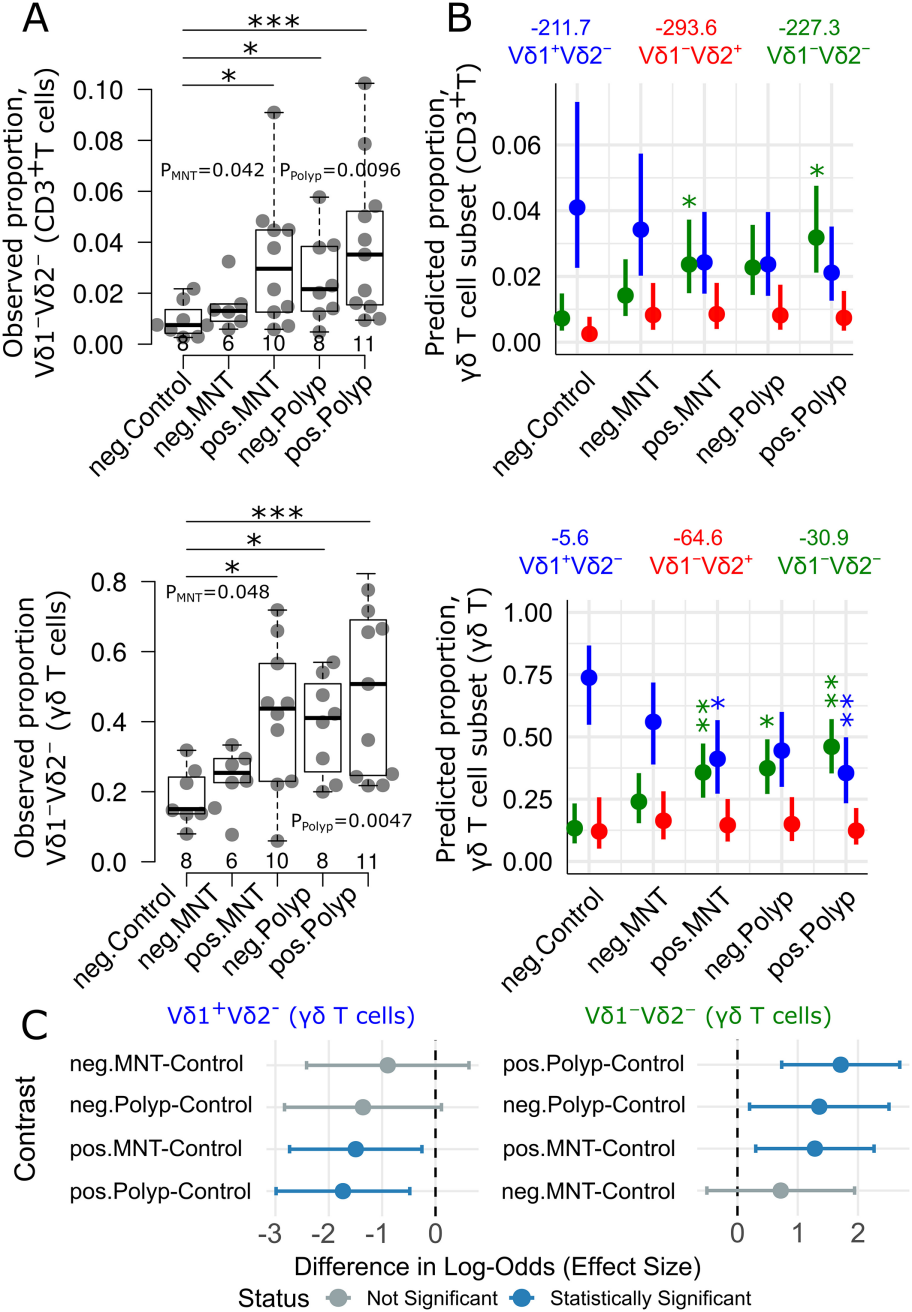
**(A)** Vδ1-Vδ2- γδ T cell subset frequencies, stratified by the presence (prefix: pos) or absence (neg) of eosinophil infiltration in different mucosal locations. The upper row shows the proportions among CD3+ T cells and the bottom row shows the proportions among γδ T cells. MNT and Polyp designate individuals with eCRSwNP. Van der Waerden test (global P-value) for independent groups (MNT vs. eosinophil-negative controls, Polyp vs. eosinophil-negative controls), followed by Van der Waerden’s many vs. one (control) *post-hoc* comparisons (single-step adjustment). Boxplots are defined by medians and their respective interquartile ranges (IQRs). Vertical lines extend to ±1.5 IQR. **(B)** Beta-regression, linear mixed model. The predicted proportions of cells are shown after accounting for sample-relatedness, age, and sex (5-level Tukey’s *post-hoc* comparisons). For simplicity, all non-significant *post-hoc* comparisons are omitted from display. An asterisk denotes a significant difference compared to controls (*P< 0.05, **P< 0.01, ***P< 0.005). For visual clarity, the data points corresponding to different color-coded cell subsets (horizontal label) are slightly offset horizontally. The colored numbers correspond to the second-order corrected Akaike information criteria for each model. The group-wise sample size is available in Panel **(A, C)** The forest plot depicting the effect size (log-odds ratio and 95% confidence interval with Tukey’s correction) for the selected comparisons from Panel **(B)** (middle row). The controls are equal to the eosinophil-negative samples. Statistically significant differences are indicated by a blue color (95% confidence intervals excluding zero).

On closer look, widespread inflammation (high LM bin) was associated with larger total γδ T cell fractions in non-lesional middle turbinate samples (**Figure 5**), which contained significantly higher numbers of Vδ1^-^Vδ2^+^ and Vδ1^-^Vδ2^-^ cells than typical nasal mucosa (**Figure 6**). In support, a progressive accumulation of Vδ1^-^Vδ2^+^, Vδ1^-^Vδ2^-^, and total γδ cells was observed as LM CT scores increased (Spearman’s partial R, age-adjusted estimates, R|_Vδ1-Vδ2+_ = 0.56, P = 0.037; R|_Vδ1-Vδ2-_ = 0.71, P = 0.00025, R|γδ = 0.73, P = 0.0023) (**Figures 7A, B**). The gain of Vδ1^-^Vδ2^-^ T cells (both absolute and relative) was particularly evident in eosinophil-positive samples (**Figure 8**). The overall number of Vδ1^+^Vδ2^-^ T cells remained unchanged (**Figures 3**, **6**, **8**). As a result, their proportional representation decreased, with more pronounced reductions observed for severe SNOT-20 scores (**Figures 7C, D**). Conversely, limited inflammation (low LM bin, MNT samples) was characterized by γδ T cell numbers and composition similar to those of healthy mucosa, although this could be underpowered due to the small number of individuals in both bins (**Figure 6**).

**Figure 9:**
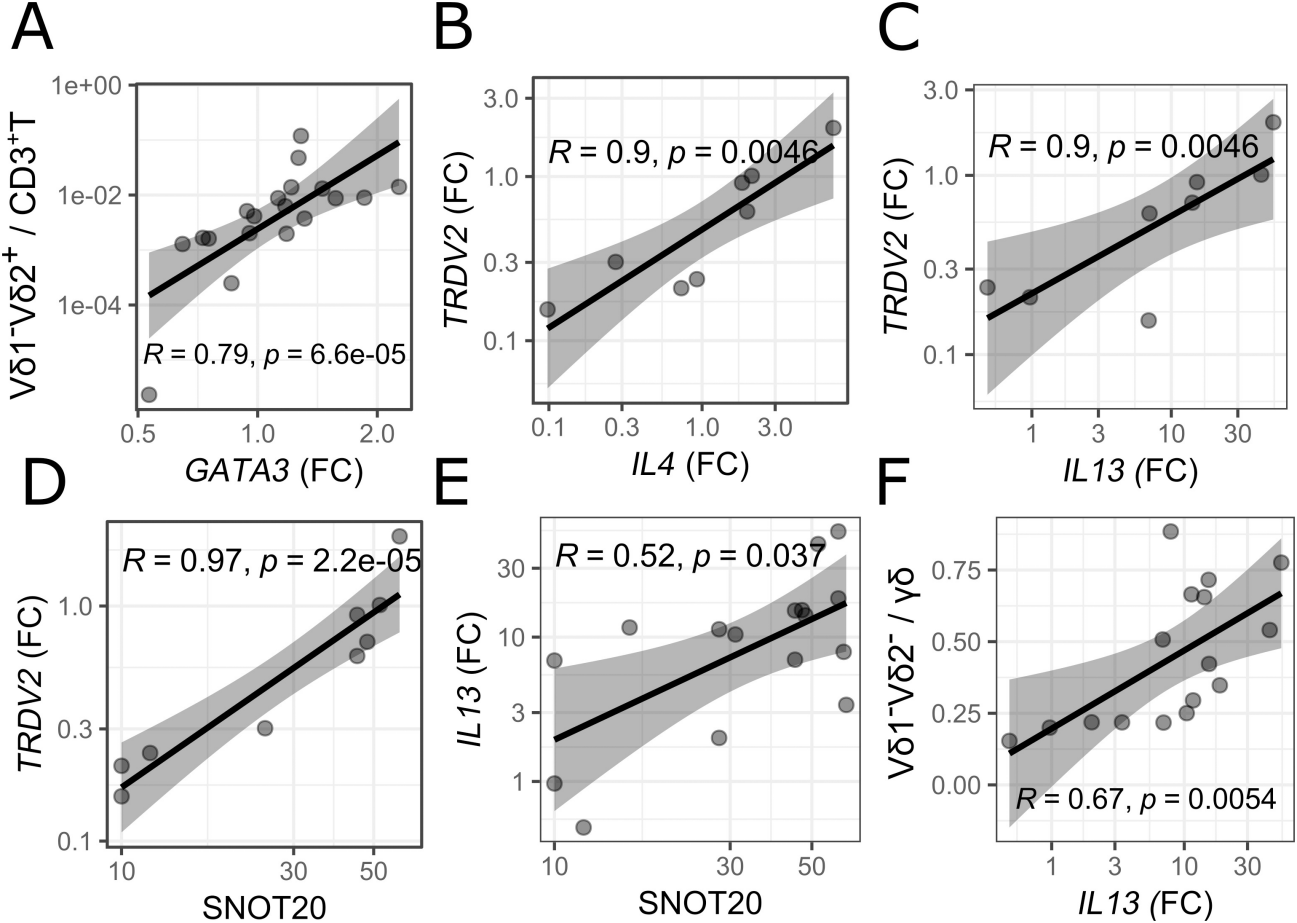
The relationship between the composition of nasal γδ T cells, disease severity [Sino-Nasal Outcome Test-20 (SNOT20)], and selected gene expression [fold change (FC)] in mucosal mononuclear cells. This analysis only included independent measurements (controls and polyps) from successful RT-qPCR experiments. Each data point represents one donor. The solid black line shows the least squares fit and the shaded ribbon corresponds to the 95% confidence interval. R and P-values belong to Spearman rank correlation test. The X- **(A–F)** and Y-axes **(A–E)** are log-scaled. *GATA3* GATA Binding Protein 3, IL Interleukin, *TRDV2* T Cell Receptor Delta Variable 2.

In polypoid mucosa, the number of Vδ1^-^Vδ2^+^ cells mirrored the findings from the middle turbinate (cosine similarity=0.895), reaching the peak (and statistically significant difference) with higher inflammatory activity (**Figure 7**). In this respect, low-grade polyps (Malm 1-2) showed more similarity to non-lesional MNT than high-grade polyps (Malm 3) (**Figure 7F**). Overall, a close correlation was found between the size of the Vδ1^-^Vδ2^+^ T cell population and the local, bulk expression of *GATA3*, a gene encoding a master regulator of the type 2 immune response (**Figure 9A**). Concurrently, an ongoing *TRDV2* transcription was observed in *IL4*/*IL13*-enriched mucosa (**Figures 9B, C**), largely in parallel with the worsening SNOT-20 scores (**Figures 9D, F**). Unlike MNT, polypoid mucosa maintained an expanded Vδ1^-^Vδ2^-^ T cell population even under low inflammatory conditions, regardless of disease activity, with and without severe inflammation (**Figure 6**). Again, Vδ1^-^Vδ2^-^ T cells were more likely to be found at higher frequencies in eosinophil-rich polyps than in eosinophil-poor polyps (**Figure 8**). Transcriptional data complement this picture, linking the local surge in Vδ1^-^Vδ2^-^ numbers with maxima in bulk RNA expression of *IL13* (**Figure 9G**), a cytokine strongly expressed by tissue eosinophils from nasal polyps, mastocytes (37), CD4^+^Th2/Tf cells (12, 38, 39), and airway ILC2 cells (40, 41). As with MNT, the total numbers of Vδ1^+^Vδ2^-^ T cells did not differ from control samples, but their relative frequency within the γδ compartment rapidly declined with deteriorating SNOT-20 scores (**Figure 7E**).

Altogether, eCRSwNP was associated with mucosal γδ T cell remodelling at multiple levels. Apparently, local γδ T cell frequencies exhibited different dynamics at lesional and non-lesional sites depending on the cell type, donor characteristics, and disease activity. Although conclusions drawn from a modest number of donors are not yet robust, the numerical and compositional observations appear to be broadly consistent across the flow cytometry and RT-qPCR data.

Nevertheless, this does not obviate the necessity of verifying the results with a larger sample. Until then, we emphasize that this modelling exercise is highly simplistic, with residual uncertainties large enough to prevent a strong test of γδ T cell kinetics in eCRSwNP. As with any descriptive study, the observed associations do not allow us to deduce causality. However, it may be useful to consider the qualitative lessons provided by this study.

## 4 Discussion

γδ T cells represent a minor but functionally versatile lymphocyte compartment within the sinonasal mucosa, where they participate in barrier surveillance, epithelial repair, and rapid cytokine responses. Here we observed coordinated numeric and compositional changes of the local γδ T cell pool across eCRSwNP nasal tissues. The most prominent features included expansion of Vδ1^-^Vδ2^+^ and double-negative (Vδ1^−^Vδ2^−^) subsets, with a relative contraction of Vδ1^+^Vδ2^-^ predominance. These compositional changes followed an ordered gradient from healthy mucosa through non-lesional middle turbinate tissue to nasal polyps and were associated with radiologic, symptomatic, and eosinophilic disease burden, suggesting progressive immune and tissue remodelling of nasal mucosa. Shifts in subset composition were accompanied by bulk mononuclear transcriptional signatures characteristic of type-2 immune polarization, reflected in correlations between *GATA3* expression and Vδ1^−^Vδ2^+^ abundance, increased *TRDV2* transcription in *IL4*/*IL13*-enriched tissues, and *IL13* upregulation paralleling expansion of Vδ1^−^Vδ2^−^ subsets. Importantly however, the RT-qPCR measurements were performed on bulk mononuclear cell preparations, hindering the cellular source of *TRDV2, IL4, IL13* and *GATA3* transcripts. Consequently, these data provide evidence of co-occurring transcriptional and immunophenotypic changes but do not establish direct functional activity of γδ T cell subsets or their contribution to cytokine production within the tissue microenvironment. At the cohort level, demographic and inflammatory features provided further context for interpreting immune findings. Although age, serum IgE, CRP, and allergy prevalence were comparable between patients and controls, the male predominance within eCRSwNP reflected epidemiologic patterns typical of polyp cohorts (42), particularly within eosinophilic endotypes (43). Histologic eosinophilia, present in over half of patients, clustered with younger age and elevated IgE and aligned with higher Lund–Mackay scores, consistent with evidence that amplified eosinophilic inflammation drives radiologic disease severity (44–46). Across tissue compartments, γδ T cell numbers and composition revealed baseline mucosal landscape but with disease-specific alterations. Healthy and non-inflamed mucosa were dominated by Vδ1^+^Vδ2^-^ and Vδ1^−^Vδ2^−^ populations, consistent with the tissue-resident Vδ1-biased γδ T cell architecture reported across adult mucosal tissues by Gray et al. (15). In healthy nasal, pulmonary and intestinal tissues, Vδ1 cells exhibit transcriptional signatures associated with tissue residency and local immune surveillance, distinguishing them from the circulating, functionally diverse Vδ2 compartment. Consistent with this biology, Vδ1^+^Vδ2^−^ cells remained a prominent component of the mucosal γδ T-cell compartment throughout disease progression. However, increasing disease severity was accompanied by marked enrichment of Vδ1^−^Vδ2^+^ and Vδ1^−^Vδ2^−^ subsets, resulting in a shift in γδ T cell composition and increased bulk γδ T cell heterogeneity within polyp tissue. Thus, the reduced proportional representation of Vδ1^+^Vδ2^−^ cells among γδ T cells appears to reflect increased representation of alternative γδ T cell subsets rather than depletion of the resident Vδ1 compartment, particularly in eosinophil-positive samples, consistent with prior reports linking γδ T cell enrichment to tissue eosinophilia, type-2 cytokine polarization, and epithelial remodelling in airway disease (19, 21, 22, 47). In line, Lee et al. (20) reported increased γδ T cell infiltration in nasal polyps and association between γδ T cell abundance and polyp recurrence, Li et al. (19) observed γδ T cell enrichment alongside type-2 inflammatory mediators in eosinophilic CRSwNP, and Yang et al. (21) identified positive correlations between Vγ1^+^ γδ T cells, eosinophil cationic protein, and metalloproteinase-7 expression. Unlike earlier work that primarily quantified total γδ T cells or Vγ-defined subsets and compared control with polyp tissue, our paired sampling approach additionally enabled analysis of non-polypoid middle turbinate mucosa from the same individuals. This revealed that γδ T-cell remodelling is already evident in non-lesional tissue and progresses along a continuum preceding overt polyp formation. The increasing representation of Vδ1^−^Vδ2^+^ and Vδ1^−^Vδ2^−^ populations suggests that previously reported increases in total γδ T cells may reflect compositional remodelling of the γδ T ell compartment rather than uniform expansion of all γδ T cell subsets. Clinically, this γδ remodelling was evident across both polypoid and non-lesional compartments. Malm grading correlated with progressive increases in total γδ cells, Vδ1^−^Vδ2^+^ and Vδ1^−^Vδ2^−^ subsets along the mucosal disease continuum. Low-grade polyps more closely resembled non-lesional mucosa, whereas high-grade lesions displayed most pronounced differences, particularly an expansion of Vδ1^−^Vδ2^−^ subset which paralleled tissue eosinophilia and higher LM scores, aligning with prior reports linking high tissue eosinophilia with poorer postoperative outcomes and increased disease burden (45, 46). Higher SNOT-20 scores were similarly associated with greater divergence of polyp immune profiles from controls along the inflammatory trajectory, and a proportional reduction in Vδ1^+^Vδ2^-^ cells. Notably, these alterations were also detectable in paired non-polypoidal, middle turbinate (MNT) mucosa, which occupied an intermediate position in principal-curve modelling, closer to controls than to polyps, consistent with early and progressive local immune remodelling preceding polyp formation. This trajectory-based γδ dynamics aligns closely with sinonasal remodelling described by recent scRNA-seq atlases (8, 10, 12), which depict eosinophilic CRSwNP as a progressive, type-2 oriented inflammation, enriched in IL-4/IL-5//IL-13 signalling, GATA3-programmed lymphocytes, and selective accumulation of type 2-skewed CD4^+^ T lymphocytes, ILC2, macrophages and dendritic cells. This immune skewing aligns with epithelial and stromal remodelling, marked by expansion of basal progenitors and secretory cells (goblet, tuft), loss of ciliated cells (12), and concurrent reprogramming of fibroblast differentiation and subset composition (8). Polyp tissue represents the apex of this remodelling gradient, maximally enriched for activated, Th2 skewed T cell states, CCL13/CCL18-secreting macrophages involved in eosinophil recruitment (12), and tissue-remodelling gene modules in epithelial and fibroblast compartments (8), while adjacent mucosa retains intermediate transcriptional imprinting reflective of field inflammation. Within this framework, the γδ gradients observed here parallel single-cell descriptions of T cell diversification in type-2–skewed niches (10). Consistently, *TRDV2* transcription was increased in *IL4/IL13*-high mucosa and correlated with worsening symptom burden (SNOT-20), while elevated local *IL13* expression was associated with expansion of Vδ1^−^Vδ2^−^ cells and higher Malm disease severity grades, particularly in eosinophil-rich middle turbinate and polyp tissue. Likewise, Vδ2^+^ abundance correlated positively with *GATA3* expression and measures of inflammatory severity. In agreement, Vδ2^+^ expansion and increased frequencies of Vδ1^−^Vδ2^−^ cells were most apparent in middle turbinate and polyp tissues with higher Lund–Mackay and Malm scores, indicating that γδ T cell remodelling is associated with more extensive local inflammation. However, because RT-qPCR was performed on unsorted mucosal mononuclear cells, the observed expression of *TRDV2, IL4, IL13*, and *GATA3* cannot be directly attributed to γδ T cell subsets. Accordingly, these findings indicate that γδ T cell remodelling, including increased Vδ1^-^Vδ2^+^ and Vδ1^−^Vδ2^−^ subset abundance in more severely affected tissues, occurs in parallel with tissue-level type-2 inflammatory signatures. However, they do not constitute evidence of γδ T cell-specific cytokine production, transcriptional programming, or effector function. In this context, previous animal studies provide a biological framework linking γδ T cells, eosinophilia and type-2 inflammation. Experimental models of allergic airway disease have demonstrated that activated γδ T cells can produce eosinophilotropic mediators (IL-5, IL-13, GM-CSF, and CCR5-binding chemokines) (48–51), and influence eosinophil recruitment, activation, and survival. In line, murine Vγ1^+^ cells have been implicated in eosinophilic recruitment through Th2 cytokine production (IL-4, IL-5, IL-13), whereas Vγ4^+^ cells appear to exert regulatory effects on airway inflammation (52). Moreover, γδ T cells have been shown to facilitate eosinophil accumulation indirectly through interactions with macrophages in murine models of endotoxin-induced pleural inflammation (53). Within our cohort, eosinophil-rich tissues showed preferential accumulation of Vδ1^−^Vδ2^−^ cells and higher total T cell fractions, indicating that γδ T cell remodelling occurs in parallel with tissue eosinophilia. However, the present study did not assess γδ T cell effector functions. Therefore, while the observed associations are reminiscent of mechanisms described in experimental models, they do not establish a direct role for γδ T cells in eosinophil recruitment or survival in eCRSwNP. Instead, γδ T-cell subset redistribution appears to occur in parallel with tissue eosinophilia and type-2 inflammation. Beyond their potential relationship with eosinophils, γδ T cells have also been implicated in broader immune networks relevant to type-2 inflammation. Functional studies suggest that γδ T cells can influence B-cell activation and immunoglobulin production (22), while human Vδ3^+^ T cells have been shown to enhance B-cell maturation and IgM secretion *in vitro* (23). Complementing these observations, ligand-receptor modelling from single-cell datasets, positions γδ T cells as potential intermediaries within myeloid-lymphoid communication networks through CCL4/CCL5–CCR1/CCR5 signalling axes. Such predicted interactions may facilitate recruitment of ALOX15^+^ cDC2 and CXCL12/19/FN1^+^ macrophages associated with Th2 differentiation and type-2 inflammation in eCRSwNP (10). Similarly, immunohistochemical studies have reported increased γδ T cell infiltration in polyp mucosa and subepithelial compartments, where their density correlates with eosinophilic infiltration (47) and local expression of IL-13, GATA3, eosinophil cationic protein, eotaxin, and metalloproteinase-7 (19, 21). Collectively, these findings place γδ T cell remodelling within the broader type-2 inflammatory landscape of eCRSwNP. However, whether γδ T cells actively contribute to disease pathogenesis or represent a response to ongoing inflammation or broader tissue remodelling remains to be determined in the future. Accordingly, the present findings are descriptive and hypothesis-generating rather than mechanistic.

The study is limited by a relatively small sample size, particularly for RT-qPCR measurements, and by a predominance of male patients, warranting cautious interpretation of our findings. Given the number of immunophenotypic, transcriptional, and clinical variables examined, some associations may be sensitive to sample size limitations and should be considered exploratory pending validation in larger independent cohorts. In addition, RT-qPCR was performed on unsorted mucosal mononuclear cells, limiting interpretation of the transcriptional data to tissue-level inflammatory signatures. Moreover, the cross-sectional design precludes causal inference, and the observed γδ T cell remodelling may represent a driver, consequence, or correlate of eCRSwNP-associated inflammation. Functional characterization of γδ T-cell subsets was also not performed. Nevertheless, the use of isolated tissue mononuclear cells rather than whole tissue homogenates enabled focused characterisation of immune-cell dynamics, while paired sampling of non-polypoid and polypoid mucosa within individual patients allowed detailed characterization of γδ T cell subsets across disease sites, revealing a progressive inflammatory continuum associated with symptom burden (SNOT-20) and disease severity (Malm scores), in line with tissue-remodelling trajectories reported by recent sinonasal scRNA-seq studies. Future studies should employ purified γδ T cell subsets for transcriptional profiling, map γδ T cell subsets and functional states at single-cell resolution, validate their tissue localization by immunohistochemistry, and use functional approaches to define the mechanistic role of γδ T cells in eCRSwNP.

## Data Availability

The raw data supporting the conclusions of this article will be made available by the authors, without undue reservation.
