# Supplementary material for: Progressive γδ T cell remodelling is associated with type-2 inflammation in eosinophilic chronic rhinosinusitis with nasal polyps
Source: Front Immunol. 2026 Jul 15;17:1857421. doi: 10.3389/fimmu.2026.1857421 (PMC13416520; doi:10.3389/fimmu.2026.1857421)
Supplement: Supplementary file 1 [file SupplementaryFile1.docx]

Supplementary Material

Supplementary Table 1. List of TaqMan probes and amplicon sizes of investigated genes.

| **Assay ID** | **Gene abbreviation** | **Gene name** | **GenBank Accesion Number** | **Amplicon size (bp)** |
| --- | --- | --- | --- | --- |
| **Hs00174122_m1** | IL-4 | interleukin 4 | NM_000589.3 | 70 |
| **Hs00174379_m1** | IL-13 | interleukin 13 | NM_002188.2 | 82 |
| **Hs00231122_m1** | GATA-3 | GATA binding protein 3 | NM_001002295.1 | 80 |
| **APGZPMN** | TRDV1 | T cell receptor delta variable 1 | X06557.1 | 212 |
| **APH6H7K** | TRDV2 | T cell receptor delta variable 2 | X53849.1 | 363 |
| **Hs99999903_m1** | ACTB1 | actin beta 1 | NM_001101.3 | 171 |

Supplementary Table 2. Overview of statistical model specifications, variable coding, and grouping structure used for downstream analyses.

| Model specifications | Equation | Factor variable/Plotted | Variable coding (Status:levels) | | |
| --- | --- | --- | --- | --- | --- |
| Suppl. Table 4. | y~Status+Age+wlogIgE+1\|ID | Status (levels) | Control |  |  |
|  |  |  | MNT(CRSwNP) | |  |
|  |  |  | Polyp(CRSwNP) | |  |
| Suppl.Table 5.B | y~Status+Age+Sex+1\|ID | Status (levels) | Control |  |  |
|  |  |  | MNT(CRSwNP) | |  |
|  |  |  | Polyp(CRSwNP) | |  |
| Fig.7. | y~Status+Age+Sex+1\|ID | Status (levels) | Control |  |  |
|  |  |  | low.LM.MNT(CRSwNP) | | |
|  |  |  | high.LM.MNT(CRSwNP) | | |
|  |  |  | low.LM.Polyp(CRSwNP) | | |
|  |  |  | high.LM.Polyp(CRSwNP) | | |
|  | y~Status+Age+Sex+1\|ID | Status (levels) | eosinophil-neg.Control | | |
| Fig.2, Fig.9. |  |  | eosinophil-neg.MNT(CRSwNP) | | |
|  |  |  | eosinophil-npos.MNT(CRSwNP) | | |
|  |  |  | eosinophil-neg.Polyp(CRSwNP) | | |
|  |  |  | eosinophil-pos.Polyp(CRSwNP) | | |
|  |  |  |  |  |  |
| ID subject's ID |  |  |  |  |  |
| Reference: Control/eosinophil-neg.Control | |  |  |  |  |
| y: Cell type proportion |  |  |  |  |  |

Supplementary Table 3. Software and algorithms (auxiliary R packages)

| **broom.mixed** | v0.2.9.5 |
| --- | --- |
| **corrplot** | v0.92 |
| **cowplot** | v1.1.1 |
| **data.table** | v1.15.4 |
| **DescTools** | v0.99.52 |
| **effectsize** | v1.0.0 |
| **extraDistr** | v1.10.0 |
| **ggbeeswarm** | v0.7.2 |
| **ggh4x** | v0.2.8.9000 |
| **ggnewscale** | v0.4.9 |
| **ggpubr** | v0.6.0 |
| **ggtext** | v0.1.2 |
| **glue** | v1.6.2 |
| **here** | v1.0.1 |
| **Hmisc** | v5.1-1 |
| **insight** | v1.4.2 |
| **kableExtra** | v1.4.0 |
| **marginaleffects** | v0.17.0 |
| **modelsummary** | v2.2.0 |
| **nlme** | v3.1-162 |
| **patchwork** | v1.3.0 |
| **performance** | v0.13.0 |
| **PMCMRplus** | v1.9.10 |
| **rstatix** | v0.7.2 |
| **scales** | v1.3.0 |
| **see** | v0.11.0 |
| **sjPlot** | v2.8.15 |
| **stats** | v4.3.1 |
| **table1** | v1.4.3 |
| **targets** | v1.8.0 |
| **tidyverse** | v2.0.0 |
| **TMB** | v1.9.17 |

Supplementary Table 4.

Supporting information, Figure 2. Generalized linear mixed model, the effect of topography, serum immunoglobulin E (IgE) levels, and age.

|  | **T (ly)** |  |  |
| --- | --- | --- | --- |
| *Predictors* | *Estimates* | *CI* | *p* |
| (Intercept) | 10.42 | 2.48 – 43.73 | **0.001** |
| Site [mnt vs. controls] | 1.81 | 0.96 – 3.42 | 0.066 |
| Site [p vs. controls] | 2.02 | 1.07 – 3.82 | **0.031** |
| wlogIgE | 0.49 | 0.29 – 0.82 | **0.006** |
| Age | 0.97 | 0.95 – 0.99 | **0.002** |
| **Random Effects** | |  |  |
| σ^2^ | 0.09 |  |  |
| τ_00_ _ID_ | 0.22 |  |  |
| ICC | 0.71 |  |  |
| N _ID_ | 31 |  |  |
| Observations | 45 |  |  |
| Marginal R^2^ / Conditional R^2^ | 0.460 / 0.846 |  |  |

wlogIgE = log-transformed, winsorized serum IgE levels, mnt middle nasal turbinate (CRSwNP) *vs.* controls, p polyps (CRSwNP) *vs.* controls. ICC intraclass correlation coefficient, R^2^ the proportion of variance explained. τ00 is the population variance of the random intercept.

Model: y~Status+Age+wlogIgE+1|ID

where Status corresponds to a factor variable with three levels (CRSwNP.MNT + CRSwNP.Polyps + Controls).

Supplementary Table 5. Beta-regression, second-order Akaike Information Criterion,

(AICc, polyps + controls).

| (A) Bootstap resampling | | | | |
| --- | --- | --- | --- | --- |
| Cell type | model1_preferred | model2_preferred |  |  |
| (parent population) | Null (constant)* | Sex+Age* |  |  |
| Vδ1^-^Vδ2^-^ (T) | 85.5 | 14.,5 |  |  |
| Vδ1^+^Vδ2^-^ (T) | 85.3 | 14.7 |  |  |
| Vδ1^-^Vδ2^+^ (T) | 55.1 | 44.9 |  |  |
| Vδ1^-^Vδ2^-^ (γδT) | 87.8 | 12.2 |  |  |
| Vδ1^+^Vδ2^-^ (γδT) | 75.3 | 24.7 |  |  |
| Vδ1^-^Vδ2^+^ (γδT) | 65.7 | 34.3 |  |  |
| *percentages based on the number of successful iterations (n=10,000) | | | | |
| (B) Beta-regression, generalized linear mixed model, cell type: Vδ1-Vδ2- (γδ) | | | | |
| Predictors | AICc | Delta_AICc | AICcWT | Complexity* |
| **Site, Age, Sex** | **-244.5** | **0** | **0.96** | **7** |
| Null (Const.) | -237.9 | 6.6 | 0.04 | 3 |
| Age, Sex | -233.87 | 10.63 | 0 | 5 |

* degrees of freedom

The Akaike weights (AICcWT) represent the probability that the candidate model is the best among the set of competing models. Bold font denotes best-fit model.
